# The non‐coding RNA OTUB1‐isoform2 promotes ovarian tumour progression and predicts poor prognosis

**DOI:** 10.1111/jcmm.13733

**Published:** 2018-07-25

**Authors:** Shunni Wang, Yan Ning, Ping Wei, Dongliag Cai, Linghui Lu, Jing Li, Yiqin Wang

**Affiliations:** ^1^ Department of Pathology Obstetrics and Gynecology Hospital of Fudan University Shanghai China; ^2^ Department of Pathology Fudan University Shanghai Cancer Center Shanghai China; ^3^ State Key Laboratory of Genetic Engineering MOE Key Laboratory of Contemporary Anthropology School of Life Sciences Fudan University Shanghai China

**Keywords:** invasion, OTUB1, OTUB1‐isoform2, ovarian cancer, proliferation

## Abstract

Ovarian cancer is the leading malignancy of the female reproductive system and is associated with inconspicuous early invasion and metastasis. We have previously reported that the oncogene OTUB1 plays a crucial role in ovarian cancer progression, but the role of its isoform, the non‐coding RNA OTUB1‐isoform2, in ovarian cancer is still elusive. Here, we reported that OTUB1‐isoform2 expression in ovarian cancer tissues was significantly higher than that in the paired paratumorous tissues (*P *<* *.01). The patients with high expression of OTUB1‐isoform2 had larger tumours than those with low expression (*P *<* *.05). The high expression of OTUB1‐isoform2 was correlated with the involvement of bilateral ovaries (*P *<* *.05), lymph node metastasis (*P *<* *.05), vascular invasion (*P *<* *.05), greater omentum involvement (*P *<* *.01), fallopian tube involvement (*P *<* *.05), advanced FIGO stages (*P *<* *.01) and recurrence (*P *<* *.01). Moreover, OTUB1‐isoform2 served as an independent negative prognostic predictor for disease‐free survival (DFS) and disease‐specific survival (DSS). Overexpression of OTUB1‐isoform2 in the ovarian cancer cells stimulated cell proliferation, migration and invasion both in vitro and in vivo. In summary, our study suggested that OTUB1‐isoform2 is a novel prognostic biomarker with independent oncogenic functions for ovarian cancer.

## 1 INTRODUCTION

Ovarian cancer is the leading malignancy of the female reproductive system on a worldwide scale.^1^, ^2^, ^3^ In the United States, ovarian cancer was the 5th most lethal malignancy in females in 2017.^1^ The 5‐year relative survival rate for ovarian cancer at early stages was 92%, which however dropped drastically to <30% at advanced stages.^1^ In China, the mortality rate of ovarian cancer is the second highest among all gynecological malignancies.^2^ Due to its inconspicuous symptoms and signs, most patients with ovarian cancer are diagnosed at advanced stages when pelvic and peritoneal dissemination has already occurred,^4^, ^5^ suggesting a highly invasive and metastatic nature of ovarian cancer and a lack of treatment methods for the patients at advanced stages. Thus, it is crucial to understand the molecular processes that underlie the progression of ovarian cancer.

Non‐coding RNAs (ncRNAs), especially those surpassing 200 nucleotides in length (long non‐coding RNAs, lncRNAs), have gained increased attention in the past few years.^6^, ^7^ Their importance in both biological functions and molecular mechanisms has been widely recognized.^7^, ^8^, ^9^ NcRNAs play key roles in cell differentiation,^8^ signal transduction^10^ and tumorigenesis.^11^, ^12^ Moreover, lncRNAs are considered to be dysregulated in gastric,^13^, ^14^ colon,^12^ renal,^15^ breast^16^ and ovarian carcinomas.^6^ Several lncRNAs have been reported to be involved in the initiation and progression of ovarian cancer.^17^, ^18^, ^19^ Therefore, the exploration of the association between ncRNAs and ovarian cancer would be of great value.

OTU domain‐containing ubiquitin aldehyde binding 1 (OTUB1) is a member of the OTU (ovarian tumour) superfamily and has two isoforms.^20^ The predominantly expressed isoform1 encodes the OTUB1 protein, which functions as a deubiquitinase.^21^, ^22^ Researchers have found that OTUB1 fulfils oncogenic functions in somatic malignancies such as breast,^23^, ^24^ gastric^25^ and prostate carcinomas.^26^ We have also found that OTUB1 promotes tumour progression with an abnormally increased expression in ovarian cancer.^25^

The other isoform, OTUB1‐isoform2 (or otubain1 alternative reading frame 1, ARF‐1) is a splice variant that generally exists in the form of a non‐coding RNA with a length of 2518 bp^20^, ^27^ in most somatic tissues. It is only in the lymphatic tissues that OTUB1‐isoform2 could encode the protein.^20^ Compared with isoform1, OTUB1‐isoform2 is expressed at a relatively lower level, and its role in human disease is ambiguous.^20^ Soares et al found that OTUB1‐isoform2 encoded the protein ARF‐1 in the lymphatic tissues to function against OTUB1. However, our previous study found that OTUB1‐isoform2 was up‐regulated in gastric cancer and exerted oncogenic functions^27^, just like its isoform OTUB1.^25^ Therefore, it remains elusive whether OTUB1‐isoform2 is implicated in ovarian cancer, and if so, what kind of role it plays in the progression of ovarian cancer.

In this study, we aimed to investigate the expression of OTUB1‐isoform2 in ovarian cancer and its potential influence on the biological functions of ovarian tumour cells, which could help identify the role of OTUB1‐isoform2 in ovarian cancer and its clinical significance.

## 2 MATERIALS AND METHODS

### 2.1 Patients

This study collected information on 114 patients who underwent surgical resection for primary ovarian carcinoma from 2003 to 2016 at Obstetrics and Gynecology Hospital of Fudan University. All histological diagnoses were independently reviewed by two pathologists in the Department of Pathology according to the WHO classifications of Tumors of Female Reproductive Organs (2014 version). The tumour grades were divided using the 3‐tier grading system in the endometrioid subtype and the two‐tier grading system in the serous subtype according to the WHO classification of 2014 version. Since the grading systems were not recommended or unified in the clear cell and the mucinous subtypes according to the WHO classification of 2014 version, the tumours of these two subtypes were not graded in our study. None of the patients received preoperative therapy. The resected tissue samples were immediately frozen in liquid nitrogen and stored at −80°C until RNA extraction. The clinicopathologic information from all patients was obtained from medical records, pathology reports and personal interviews. The clinical information included age, gender, disease‐free survival (DFS), disease‐specific survival (DSS), surgical records and gross pathological descriptions. The staging criteria were defined according to the FIGO (International Federation of Gynecology and Obstetrics) classification system. The patients were followed‐up every 3 months during the first year after surgery and every 6 months thereafter until 31 January 2018. All patients had complete follow‐up information. DFS was calculated from the date of surgery to the date of disease recurrence (local and/or distal tumour recurrence). DSS was defined as the length of time between surgery and the disease‐specific death of the patient. The study was approved by the ethics committee of Obstetrics and Gynecology Hospital of Fudan University. Written informed consent was obtained from all participants for the use of their tissues in this study.

### 2.2 RNA isolation, reverse transcription and quantitative real‐time PCR (qRT‐PCR)

Total RNA was extracted from tissue samples and cell lines using TRIzol reagent (Invitrogen, Carlsbad, CA, USA) according to the manufacturer's protocol. Reverse transcription (RT) and quantitative real‐time PCR (RT‐qPCR) kits (Takara, Dalian, China) were utilized to evaluate the mRNA expression levels of target genes. RT and RT‐qPCR were performed as previously described.^27^ Specific primer sets for OTUB1 and OTUB1‐isoform2 were designed and described in the previous study.^27^ PCR of both genes and sequencing of the PCR products were previously performed to ensure specificity.^27^ β‐ACTIN was used as an endogenous control to normalize the data. The primer sequences for OTUB1, OTUB1‐isoform2, E‐CADHERIN and N‐CADHERIN were also previously listed.^27^ The other primer sequences were as follows: TTTCTCCACGCCTCCAGTT (forward) and ATGTCTTCGGCCAGGTTGT (reverse) for VIMENTIN; GGCAGGTCATCACCATCGG (forward) and CGTGTTGGCGTAGAGGTCTTT (reverse) for β‐ACTIN. For statistical analyses, the levels of OTUB1‐isoform2 were divided into low and high expression groups by the median value as established in previous studies.^13^, ^28^

### 2.3 Cell lines and culture conditions

Human HEK‐293FT cells and the human ovarian cancer cell lines A2780, SKOV3, CAOV3, ES‐2, HO8910, 3AO and NIH: OVCAR‐3 were purchased from Fudan IBS Cell Bank and Type Culture Collection of Chinese Academy of Science. The cells were cultured in RMPI‐1640 or high‐glucose DMEM (Gibco, Carlsbad, CA, USA) supplemented with 10% fetal bovine serum (FBS; Gibco, Carlsbad, CA, USA) and 1% penicillin and streptomycin (Sigma, St. Louis, MO, USA). All cell lines were maintained at 37°C and 5% CO_2_ in a humidified atmosphere.

### 2.4 Plasmid transfection and Lentivirus transduction

The pcDNA3.1‐OTUB1 and pcDNA3.1‐OTUB1‐isoform2 plasmids were previously constructed and described in our previous studies.^27^, ^29^ Both plasmids were verified by both sequencing of PCR products and RNA electrophoresis.^27^ For the lentivirus transduction, virus particles were harvested 48 hours after co‐transfection of the pHBLV‐IRES‐ZsGreen‐PGK‐puro constructs, the packaging plasmid ps‐PAX2 and the envelope plasmid pMD2G into HEK‐293FT cells using Lipofectamine 3000 Reagent (Life Technologies, Carlsbad, CA, USA) according to the manufacturer's instructions. A2780 and SKOV3 cells were infected with recombinant lentivirus‐transducing units (pHBLV‐OTUB1‐isoform2 or empty vector) plus 6‐μg/mL polybrene (Sigma, St. Louis, MO, USA). After 72 hours of incubation, the cells were harvested for the assays described below. The stable infection of cells was maintained with 2 μg/mL of puromycin (Sigma, St. Louis, MO, USA).

### 2.5 Antibodies and reagents

The following antibodies were used in this study for western blot and were purchased from Cell Signaling Technology (Boston, MA, USA): β‐ACTIN (8H10D10, #3700), OTUB1 (D8F7, #3783), E‐CADHERIN (24E10, #3195), N‐CADHERIN (D4R1H, #13116), VIMENTIN (D21H3, #5741), P21 (12D1, #2947) and CCNB1 (V152, #4135). The following reagents were used: Lipofectamine 3000 transfection reagent (Lot. 11668‐027 Invitrogen, Carlsbad, CA, USA) and RIPA lysis buffer (Lot. 89901, Thermo Scientific, USA).

### 2.6 Cell proliferation assays

Cell proliferation was evaluated using a Cell Counting Kit‐8 (CCK‐8) (Dojindo, Kumamoto, Japan), an EdU (5‐Ethynyl‐2′‐dexoyuridine) DNA immunofluorescence kit (Life Technology, Carlsbad, CA, USA) and a colony formation assay. The former two assays were performed using 2 × 10^3^ A2780 and SKOV3 cells transfected with pcDNA3.1 or pcDNA3.1‐OTUB1‐isoform2 in 96‐well plates according to the recommended protocols.

For the CCK‐8 assay, an automatic microplate reader (Synergy H1, Biotek, VT, USA) was used to determine the absorbance at 450 nm.

For the EdU immunofluorescence assay, EdU is a thymidine mimicry and could penetrate DNA during DNA replication. By staining EdU with a specific immunofluorescence dye, EdU‐positive cells represent living cells in the S phase, reflecting the proliferating activity of the cells. Images were obtained at 100× and counted at 200× under an immunofluorescence microscope (Olympus, Tokyo, Japan).

In the plate colony formation assay, A2780 and SKOV3 cells were stably infected with Lenti‐NC or Lenti‐OTUB1‐isoform2. Then, 800 cells were seeded onto 6‐well plates and incubated for 2 weeks, followed by ethanol fixation and crystal violet staining. The number of colonies with more than 30 cells was counted.

### 2.7 Cell motility and invasion assays

A wound‐healing assay was performed to assess cell motility. Transfected cells were plated at equal density in 6‐well plates and grown to 90% confluence. Cells were pretreated with Mitomycin C (Sigma, St. Louis, MO, USA) for 1 hour at 37°C to exclude the potential interference of cell proliferation. Wounds were scratched with sterile pipette tips: loose cells were then rinsed with PBS, and serum‐free medium was added. The wound closing procedure was observed at 40× under a microscope (BX43, Olympus, Japan) at two time points: one was just after the initial scratch, and the other was at 24 hours post‐scratch.

The Transwell chambers (8 μm, 24‐well format) (Corning Ltd. Co., USA) were used for the cell invasion assay. 4 × 10^4^ cells in 100 μL of serum‐free medium were loaded onto the upper inserts, and 500 μL of culture medium containing 10% FBS was loaded into the lower chambers as a chemo‐attractant. After a 24‐hour incubation period at 37°C, the cells that had migrated through the filters were fixed in ethanol and stained with crystal violet. Photographs were taken at 200× under microscope (BX43, Olympus, Japan), and the number of invaded cells was counted at 400×.

### 2.8 Western blot

Cells were lysed in RIPA buffer (Sigma, St. Louis, MO, USA) supplemented with a protease inhibitor and a phosphatase inhibitor (Roche, Basel, Switzerland). The protein concentration was measured using a BCA protein assay kit (Thermo Scientific, USA). Isolated proteins were probed with the indicated primary antibodies, followed by incubation with HRP‐linked secondary antibodies and detection using the ECL system (Thermo Fisher, USA). β‐ACTIN was used as an endogenous control.

### 2.9 Immunohistochemistry

Consecutive paraffin sections of the livers of nude mice were deparaffined in a series of xylene baths and then rehydrated using a graded alcohol series. Sections were subjected to steam heat‐induced epitope retrieval in the presence of 10 mmol/L sodium citrate buffer (pH 6.0) for 5 minutes, and were incubated overnight at 4°C with primary antibodies against E‐CADHERIN, N‐CADHERIN and VIMENTIN at a 1:200 dilution. Tissues were then incubated with a biotinylated secondary antibody. The avidin‐biotin complex/HRP (ABC/HRP) was used along with the DAB chromogen to visualize protein expression.

### 2.10 In vivo nude mouse xenograft and tumour metastasis models

The Shanghai Medical Experimental Animal Care Commission approved the animal experiments. Female BALB/c‐nu mice (6 weeks of age, 18‐20 g) were maintained under specific pathogen‐free conditions at the Fudan University Experimental Animal Department. All experimental procedures involving animals were undertaken in accordance with the institute's guidelines. For the xenograft models, a total of 1 × 10^7^ SKOV3 cells stably infected with Lenti‐NC or Lenti‐OTUB1‐isoform2 were infected subcutaneously (s.c.) in the flank regions of nude mice (n = 4 per group). The tumour sizes were recorded every 2 days for 30 days. For the intraperitoneal tumour metastasis models, 8 × 10^6^ SKOV3 cells stably infected with Lenti‐NC or Lenti‐OTUB1‐isoform2 were infected intraperitoneally (i.p.) (n = 3 per group). The abdominal circumferences of the mice were measured every 2 days. All the mice were anaesthetized and sacrificed. The xenografts were excised and measured. The tumour volumes were calculated using the formula 1⁄2 × *r*1^2^ × *r*2 (*r*1 < *r*2). The intraperitoneal organs (eg livers, peritoneal membranes and intestines, etc.) were excised and examined for the implanted lesions followed by sampling. All the tissues were fixed and embedded with paraffin. Hematoxylin‐eosin staining was used to observe the lesions through a microscope. Immunochemical staining was used to detect the expression of E‐CADHERIN, N‐CADHERIN and VIMENTIN in the metastatic xenografts.

### 2.11 Statistical analysis

All statistical analyses were performed using SPSS 20.0 (IBM, Chicago, IL, USA) and GraphPad Prism 7. The chi‐square test and the Fisher's exact probability test were used to analyse the correlations between OTUB1‐isoform2 expression and the clinicopathologic parameters. The Student's *t* test and one‐way ANOVA were used to analyse the results of two or multiple groups. Disease‐free survival (DFS) rate was calculated from the date of surgery to the date of local and/or distal recurrence. Disease‐specific survival (DSS) rate was defined as the length of time between the diagnosis and death or the end of follow‐up. DFS and DSS were calculated with the Kaplan–Meier method and analysed with the log‐rank test. Variables with a value of *P *<* *.05 in the univariate analysis were used in the multivariate analysis on the basis of the Cox proportional hazards model. All the experiments were repeated for at least three times. All the tests were two‐sided, and *P* value <.05 was considered statistically significant.

## 3 RESULTS

### 3.1 OTUB1‐isoform2 was abnormally increased in ovarian cancer and was correlated with poor prognosis

**Figure 1:**
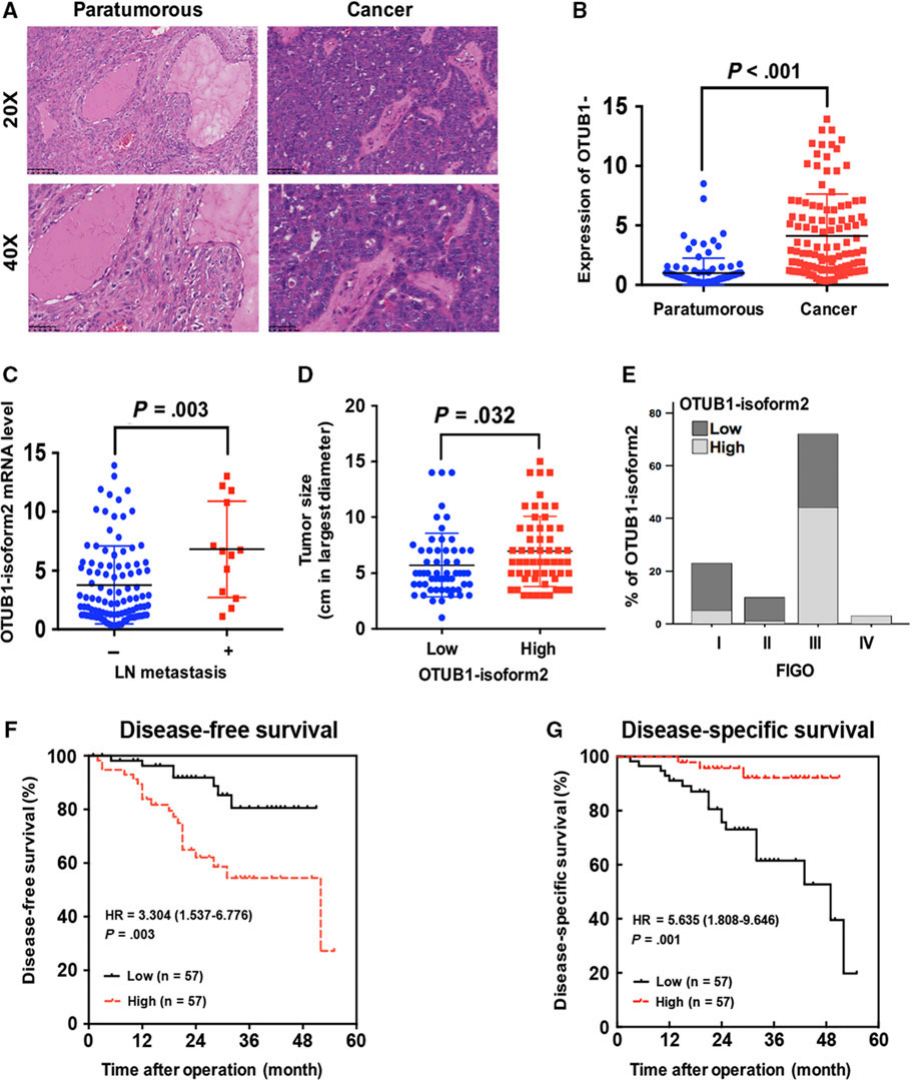
OTUB1‐isoform2 is increased in ovarian cancer tissues and is correlated with poor prognosis. A, The Hematoxylin‐Eosin staining of paratumorous and tumour tissues under the microscope. Up: scale bars = 100 μm. Bottom: scale bars = 50 μm. B, The expression of OTUB1‐isoform2 in paratumorous and ovarian cancer tissues was determined by RT–qPCR. β‐ACTIN was used as an endogenous control to normalize the data. C, The expression of OTUB1‐isoform2 in cases with or without lymph node(LN) metastasis was determined by RT–qPCR. β‐ACTIN was used as an endogenous control to normalize the data. D, The tumour sizes in low and high expression groups of OTUB1‐isoform2 were analysed using Student's *t* test. The *P* value was illustrated on the graph. E, The expression of OTUB1‐isoform2 in different FIGO stages was determined by the RT‐qPCR. β‐ACTIN was used as an endogenous control to normalize the data. F‐G, Kaplan‐Meier curves for DFS (F) and DSS (G) of patients with ovarian cancer based on OTUB1‐isoform2 expression

Since OTUB1‐isoform2 exists as a ncRNA in somatic organs except in the lymphatic tissues (https://www.ncbi.nlm.nih.gov/nuccore/NR_003089.1)%5b20 20, and our previous study has also confirmed its existence as a ncRNA in gastric cancer,^27^ we first determined the level of OTUB1‐isoform2 mRNA using RT‐qPCR with the specific primers. The RT‐qPCR assay was performed in paired tumour and paratumorous tissues from 114 patients with ovarian cancer (Figure 1A). The specificities of the primers were identified in our previous study.^27^ We found that the OTUB1‐isoform2 mRNA level was significantly higher in the 114 malignant lesions than in the paired paratumorous areas (*P *<* *.001, Figure 1B), suggesting that OTUB1‐isoform2 could be detected in ovarian cancer and that its mRNA level is abnormally increased.

Next, we analysed the correlation between OTUB1‐isoform2 expression and the clinicopathologic status of patients with ovarian cancer. We found that the OTUB1‐isoform2 mRNA level was statistically higher in patients with lymph node metastasis than in those without lymph node metastasis (*P *=* *.003, Figure 1C). When OTUB1‐isoform2 was divided into the high and low expression groups by the median value according to previous studies,^9^, ^28^ we found that the tumour size in the high expression group was significantly greater than that in the low expression group (*P *=* *.032, Figure 1D). High expression of OTUB1‐isoform2 was significantly correlated with the involvement of bilateral ovaries (*P *=* *.023, Table 1), positive lymph node metastasis (*P *=* *.039, Table 1), positive vascular invasion (*P *=* *.031, Table 1), greater omentum involvement (*P *=* *.005, Table 1), fallopian tube involvement (*P *=* *.038, Table 1) and recurrence (*P *=* *.002, Table 1). More importantly, the proportion of high expression of OTUB1‐isoform2 increased with advancement in FIGO stage (Figure 1E). High expression of OTUB1‐isoform2 was tightly correlated with advanced FIGO stages (*P *<* *.01, Table 1). But the expression of OTUB1‐isoform2 was not correlated with the histological subtypes of ovarian cancer (high grade serous, low grade serous, clear cell and mucinous types) (Table 1). In the endometrioid and serous subtypes, the expression of OTUB1‐isoform2 was not correlated with the tumour grades (Table 1).

**Table 1:** Relationship between OTUB1‐isoform2 and pathological factors of ovarian cancer

Characteristics	Number of case	%	OTUB1‐isoform2		χ^2^	*P* c
			Low	High		
Age (y)						
≤50	53	46.49	27	26	0.035	.851
>50	61	53.51	30	31		
Ovary involvement						
Left	35	30.70	24	11	7.528	.023^d^
Right	32	28.07	15	17		
Bilateral	47	41.23	18	29		
Histology						
High grade serous	65	57.02	27	38	6.043^a^	.196
Low grade serous	5	4.39	3	2		
Endometrioid	18	15.79	9	9		
Clear cell	20	17.54	14	6		
Mucinous	6	0.06	4	2		
Tumour grades (endometrioid subtype)						
1	10	55.56	4	6	2.670^a^	.263
2	5	27.77	4	1		
3	3	16.67	1	2		
Tumour grades (serous subtype)						
Low	5	7.14	3	2	0.738^b^	.645
High	65	92.86	27	38		
Lymph node metastasis						
−	101	88.60	54	47	4.254	.039^d^
+	13	11.40	3	10		
Vascular invasion						
−	98	85.96	53	45	4.653	.031^d^
+	16	14.04	4	12		
Greater omentum involvement						
−	57	54.81	35	22	7.717	.005^d^
+	47	45.19	16	31		
Fallopian tube involvement						
−	51	44.74	31	20	4.293	.038^d^
+	63	65.26	26	37		
FIGO stage						
I	24	21.05	19	5	21.989^a^	<.001^d^
II	11	9.65	9	2		
III	76	66.67	29	47		
IV	3	2.77	0	3		
FIGO staging						
I + II	38	33.33	30	8	19.105	<.001^d^
III + IV	76	66.67	27	49		
Recurrence						
−	86	75.44	50	36	9.279	.002^d^
+	28	24.56	7	21		

a Likelihood ratio.

b Correction for continuity.

c All statistical tests were two‐sided. Significance level: *P *<* *.05.

d *P *<* *.05.

We then conducted a Kaplan–Meier analysis using the log‐rank test to explore the influence of OTUB1‐isoform2 expression on patient survival. The total median follow‐up time for the patients who were still alive at the endpoint for analysis was 55 months. The median follow‐up time for the patients who were still alive at the endpoint was 24.50 months in the high OTUB1‐isoform2 expression group and 28.00 months in the low expression group. The results showed that patients with high OTUB1‐isoform2 expression (n = 57) had significantly shorter DFS (*P *=* *.003; Figure 1F) and DSS rates (*P *=* *.001; Figure 1G) compared with those with low expression (n = 57). A univariate Cox analysis showed that lymph node metastasis, FIGO stage and OTUB1‐isoform2 expression were correlated with the DFS and DSS rates (*P *<* *.05, Table 2, 3). A multivariate analysis using the Cox proportional hazards model further demonstrated that the OTUB1‐isoform2 level was an independent predictor of both DFS (*P *=* *.042, Table 2) and DSS (*P *=* *.046, Table 3) along with FIGO stage (*P *=* *.041 for DFS Table 2; *P *=* *.009 for DSS Table 3). These results show that the abnormally increased OTUB1‐isoform2 expression in ovarian cancer predicts poor survival and is a prognostic biomarker for the disease.

**Table 2:** Univariate and multivariate analyses of clinicopathological factors for disease‐free survival (DFS) in ovarian cancer

Variable	Univariate analysis		Multivariate analysis	
	HR (95% CI)	*P* a	HR (95% CI)	*P* a
Age (<50/≥50)	0.830 (0.390‐1.770)	.630		
Vascular invasion (present/absent)	1.318 (0.499‐3.482)	.578		
The greater omentum invasion (present/absent)	1.691 (0.790‐3.618)	.176		
Fallopian tube involvement (present/absent)	1.245 (0.588‐2.634)	.567		
Lymph node metastasis (present/absent)	3.373 (1.421‐8.005)	.006^b^		
FIGO stage (III + IV/I + II)	2.374 (1.238‐4.552)	.009^b^	2.535 (1.039‐6.183)	.041^b^
OTUB1‐isoform2 (high/low)	3.425 (1.446‐8.117)	.005^b^	1.967 (1.025‐3.775)	.042^b^

HR, Hazard ratio; CI, confidence interval.

a All statistical tests were two‐sided. Significance level: *P *<* *.05.

b *P *<* *.05.

**Table 3:** Univariate and multivariate analyses of clinicopathological factors for disease‐specific survival(DSS) in ovarian cancer

Variable	Univariate analysis		Multivariate analysis	
	HR (95% CI)	*P* a	HR (95% CI)	*P* a
Age (<50/≥50)	0.818 (0.346‐1.937)	.648		
Vascular invasion (present/absent)	1.690 (0.617‐4.632)	.307		
The greater omentum invasion (present/absent)	2.292 (0.920‐5.713)	.075		
Fallopian tube involvement (present/absent)	2.144 (0.860‐5.343)	.102		
Lymph node metastasis (present/absent)	2.999 (1.162‐7.742)	.023^b^		
FIGO stage (III + IV/I + II)	5.369 (1.856‐15.526)	.002^b^	3.999 (1.412‐11.327)	.009^b^
OTUB1‐isoform2 (high/low)	5.883 (1.731‐20.001)	.005^b^	3.549 (1.020‐12.347)	.046^b^

HR, Hazard ratio; CI, confidence interval.

a All statistical tests were two‐sided. Significance level: *P *<* *.05.

b *P *<* *.05.

### 3.2 OTUB1‐isoform2 was expressed independently of OTUB1

Since we previously found that OTUB1, the predominant isoform of this gene, is up‐regulated in ovarian cancer,^29^ it is essential to exclude the possibility that the expression of OTUB1‐isoform2 is dependent on its predominant isoform, OTUB1. First, we used specific primers that were previously designed for each isoform.^20^, ^27^ The regular PCR products of these primers have been subjected to RNA agarose gel electrophoresis and the followed the sequencing in our previous study so that their specificities were ensured.^27^

**Figure 2:**
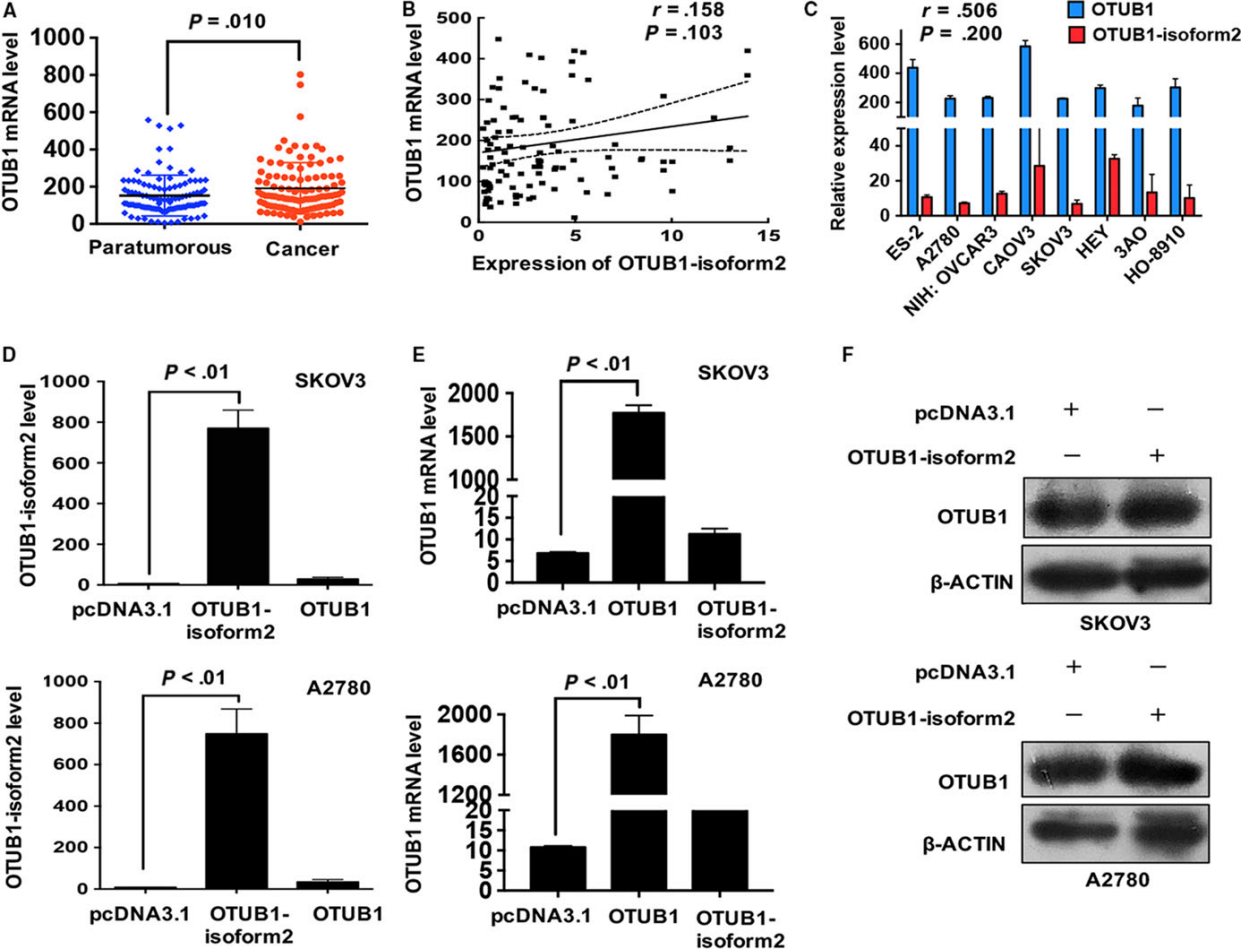
OTUB1‐isoform2 overexpression is not correlated with OTUB1 expression. A, The expression of OTUB1 in the paratumorous and cancer tissues was determined by RT–qPCR. β‐ACTIN was used as an endogenous control to normalize the data. B, The correlation between OTUB1‐isoform2 expression and OTUB1 expression in the ovarian cancer tissues was evaluated by the Pearson correlation analysis. C, The mRNA expression levels of OTUB1‐isoform2 and OTUB1 in the ovarian tumour cells were evaluated by RT–qPCR. β‐ACTIN was used as an endogenous control to normalize the data. The correlation between the two isoforms in the ovarian tumour cells was evaluated by the Pearson correlation analysis. D, The mRNA expression levels of OTUB1‐isoform2 and OTUB1 in A2780 and SKOV3 cells overexpressing OTUB1 were evaluated by RT–qPCR. β‐ACTIN was used as an endogenous control to normalize the data. E, The mRNA expression levels of OTUB1‐isoform2 and OTUB1 in A2780 and SKOV3 cells overexpressing OTUB1‐isoform2 were evaluated by RT–qPCR. β‐ACTIN was used as an endogenous control to normalize the data. F, The protein expression level of OTUB1 in A2780 and SKOV3 cells overexpressing OTUB1‐isoform2 was evaluated by western blotting; β‐ACTIN was used as a reference. Data are shown as the mean SD of three replicates

The results showed that OTUB1 mRNA expression was significantly increased in the tumour tissues compared with the paired paratumorous areas (*P *=* *.010, Figure 2A). The mRNA levels of both isoforms were statistically irrelevant in the ovarian tumour tissues according to the Pearson correlation analysis (*r* = .158, *P *=* *.103, Figure 2B). Next, we detected the mRNA levels of both isoforms in eight ovarian cancer cell lines and found that the expressions of both isoforms were irrelevant (*r* = .506, *P *=* *.200). Finally, we investigated the expression of one isoform when the other was overexpressed in the two ovarian cancer cell lines A2780 and SKOV3 which expressed relatively low levels of both OTUB1 and OTUB1‐isoform2. The overexpression of OTUB1 significantly increased the mRNA level of OTUB1 but failed to elevate the mRNA level of OTUB1‐isoform2 (*P *<* *.01, Figure 2D). Consistently, the overexpression of OTUB1‐isoform2 significantly increased the mRNA level of OTUB1‐isoform2 but failed to elevate OTUB1 at either the mRNA (*P *<* *.01, Figure 2E) or the protein level (Figure 2F). Taken together, these results suggest that the expression of OTUB1‐isoform2 is independent of its isoform OTUB1 in ovarian cancer.

### 3.3 OTUB1‐isoform2 promoted ovarian cancer cell proliferation

To investigate the potential effect of OTUB1‐isoform2 on the pathogenesis of ovarian cancer, we measured the baseline mRNA levels of OTUB1‐isoform2 in eight ovarian cancer cell lines (Figure 2C). The RT‐qPCR data showed that the OTUB1‐isoform2 mRNA levels were relatively low in all the ovarian cancer cell lines, especially in A2780 and SKOV3 cells (Figure 2C), which led to our strategy of exogenous overexpression via plasmid or lentivirus. The overexpression efficiency of the OTUB1‐isoform2 plasmid was confirmed before the RT‐qPCR experiments in A2780 and SKOV3 cells (Figure 2E).

**Figure 3:**
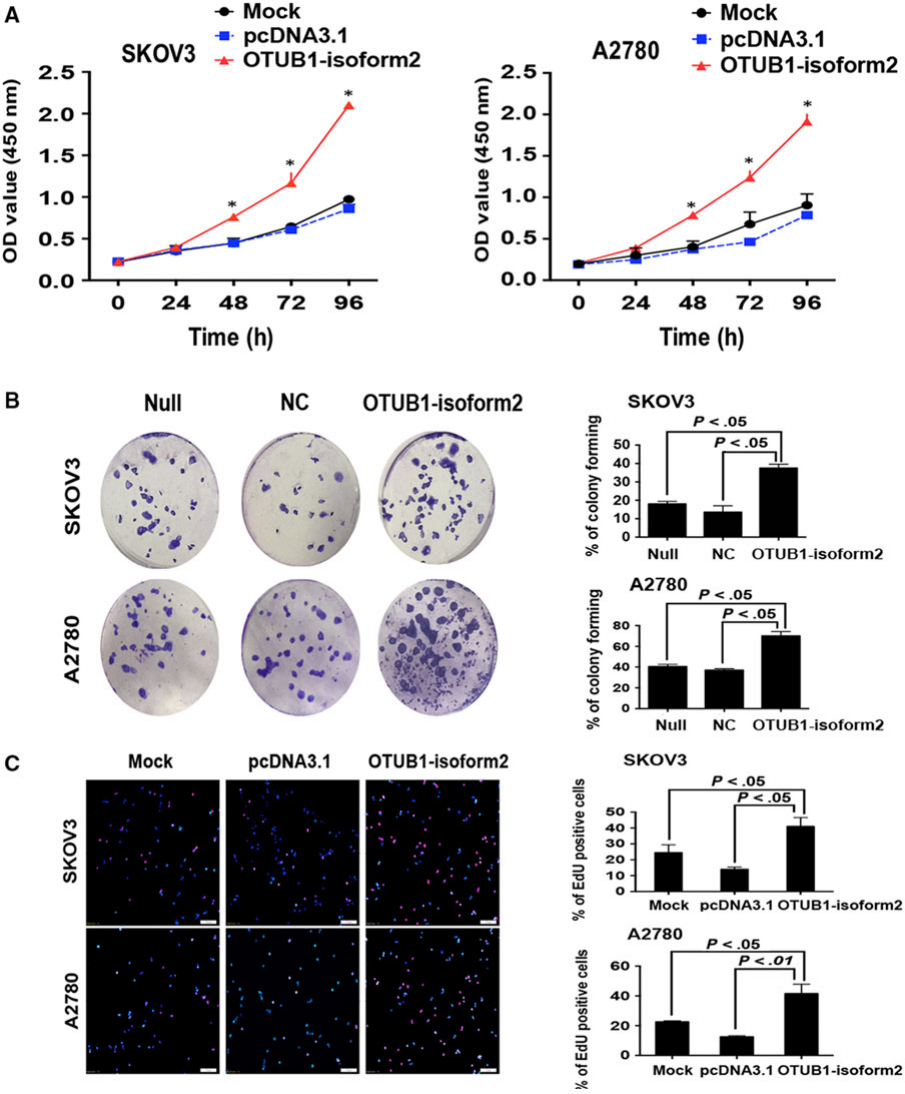
OTUB1‐isoform2 promotes ovarian tumour cell proliferation. A, Cell viability of SKOV3 and A2780 cells transfected with OTUB1‐isoform2 or pcDNA3.1 control was measured using a CCK8 assay. Data are shown as the mean SD of three replicates. *P <* *.01. B, The cell colony formation ability of SKOV3 and A2780 cells infected with Lentivirus‐OTUB1‐isoform2 or negative control was measured. Data are shown as the mean SD of three replicates. The *P* value was illustrated. C, The EdU incorporation was evaluated using an EdU imaging kit. Quantitative analysis showed EdU‐positive cells in A2780 and SKOV3 cells transfected with OTUB1‐isoform2 or pcDNA3.1 control. Cells were photographed under the immunofluorescence microscope (scale bars = 100 μm). The *P* value was illustrated

Next, we performed CCK8, colony formation and EdU (5‐Ethynyl‐2′‐deoxyuridine) immunofluorescence assays in A2780 and SKOV3 cells to observe the influence of OTUB1‐isoform2 overexpression on ovarian cancer cell proliferation. In the CCK8 assay, we found that the absorbance of the OTUB1‐isoform2‐overexpressing group was significantly higher than that of either the mock or the pcDNA3.1 control from 48 hours after transfection to the end of the measurement period in both A2780 and SKOV3 cells, suggesting that overexpression of OTUB1‐isoform2 elevated the accumulation of living ovarian tumour cells (*P *<* *.01, Figure 3A). Next, the colony formation assays showed that more colonies were formed in the OTUB1‐isoform2‐overexpression group than those in either the null or the negative control group in both A2780 and SKOV3 cells (*P *<* *.05, Figure 3B). Finally, we performed an EdU immunofluorescence assay. EdU is a thymidine (T) mimicry that can substitute for thymidine and penetrate DNA molecules during the DNA replication, thus reflecting the proliferating activities of cells (see Materials and Methods). In our study, the EdU immunofluorescence assay showed that more A2780 and SKOV3 cells in the OTUB1‐isoform2‐overexpressing group were in the S phase than those in either the mock or the pcDNA3.1 control group (*P *<* *.05, Figure 3C). Taken together, these results suggest that OTUB1‐isoform2 promotes ovarian tumour cell proliferation.

### 3.4 OTUB1‐isoform2 promoted ovarian cell migration and invasion

**Figure 4:**
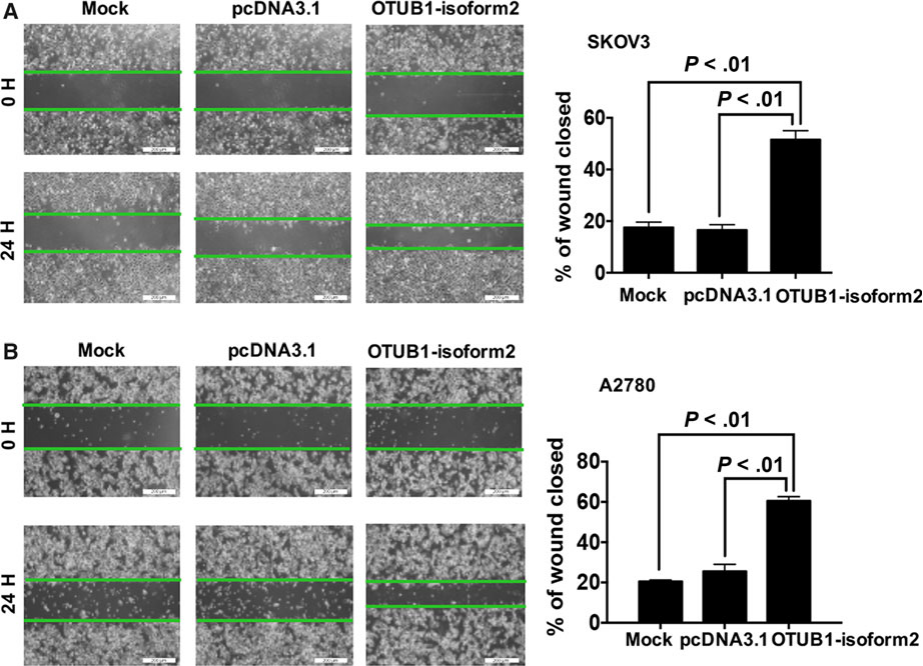
OTUB1‐isoform2 promotes ovarian tumour cell migration. A, Cell migration ability was evaluated using a wound‐healing assay; images of SKOV3 cells transfected with OTUB1‐isoform2 or pcDNA3.1 control were taken at 0 and 24‐h post‐scratch. The percentage of closed distances of healed wounds after 24 h was calculated under the microscope (scale bars = 200 μm). Data are shown as the mean SD of three replicates. The *P* value was illustrated. B, Cell migration ability was evaluated using a wound‐healing assay; images of A2780 cells transfected with OTUB1‐isoform2 or pcDNA3.1 control were taken at 0 and 24‐h post‐scratch. The percentage of closed distances of healed wounds after 24 h was calculated under the microscope (scale bars = 200 μm). Data are shown as the mean SD of three replicates. The *P* value was illustrated

**Figure 5:**
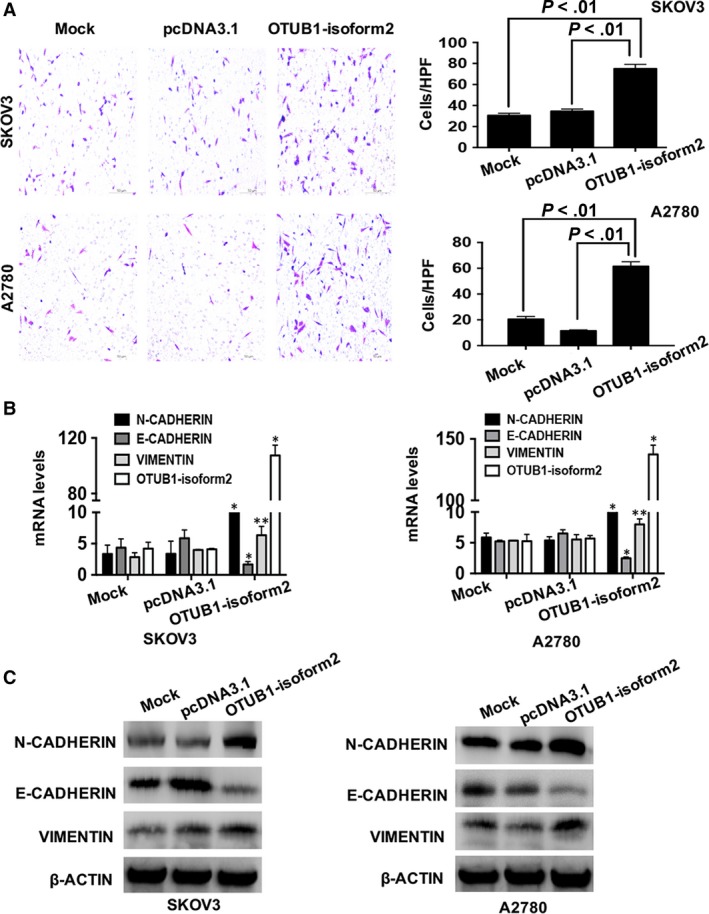
OTUB1‐isoform2 promotes ovarian tumour cell invasion. A, Cell invasion ability was evaluated using the Transwell assay in A2780 and SKOV3 cell transfected with OTUB1‐isoform2 or pcDNA3.1 control. The penetrated cells were photographed under the microscope (scale bars = 50 μm). Data are shown as the mean SD of three replicates. The *P* value was illustrated. B, The RT‐qPCR analysis of the expression levels of E‐CADHERIN, N‐CADHERIN, VIMENTIN and OTUB1‐isoform2 in SKOV3 and A2780 cells transfected with OTUB1‐isoform2 or pcDNA3.1 control. β‐ACTIN was used as a reference. Data are shown as the mean SD of three replicates; *P <* *.01, **P <* *.05. C, Western blotting analysis of the expression levels of E‐CADHERIN, N‐CADHERIN and VIMENTIN. β‐ACTIN was used as a reference. Data are shown as the mean SD of three replicates

To determine whether OTUB1‐isoform2 promotes ovarian cancer cell invasion and migration, we performed the wound healing and Transwell assays. In the wound healing assay, we found that the overexpression of OTUB1‐isoform2 accelerated the wound healing rates in both SKOV3 cells (*P *<* *.01, Figure 4A) and A2780 (*P *<* *.01, Figure 4B) compared with the mock and the pcDNA3.1 control. Consistently, the Transwell assay showed that more invaded SKOV3 (*P *<* *.01, Figure 5A) and A2780 cells (*P *<* *.01, Figure 5B) were counted in the OTUB1‐isoform2‐overexpressing group than in either the mock or the pcDNA3.1 control group. Finally, we detected the expressions of the invasion‐related genes E‐CADHERIN, N‐CADHERIN and VIMENTIN.^30^, ^31^ The invasion‐related genes’, N‐CADHERIN and VIMENTIN, expressions were elevated in the OTUB1‐isoform2‐overexpressing A2780 and SKOV3 cells when compared with either the mock or the pcDNA3.1 control both at the mRNA (*P *<* *.05, Figure 5B) and protein levels (Figure 5C). In contrast, the expression of E‐CADHERIN, which is inversely associated with invasion, decreased in the OTUB1‐isoform2‐overexpressing A2780 and SKOV3 cells when compared with either the mock or the pcDNA3.1 control both at the mRNA (*P *<* *.01, Figure 5B) and protein levels (Figure 5C). Collectively, these results suggest that OTUB1‐isoform2 enhances ovarian tumour cell migration and invasion.

### 3.5 OTUB1‐isoform2 promoted ovarian tumour growth and metastasis in vivo

**Figure 6:**
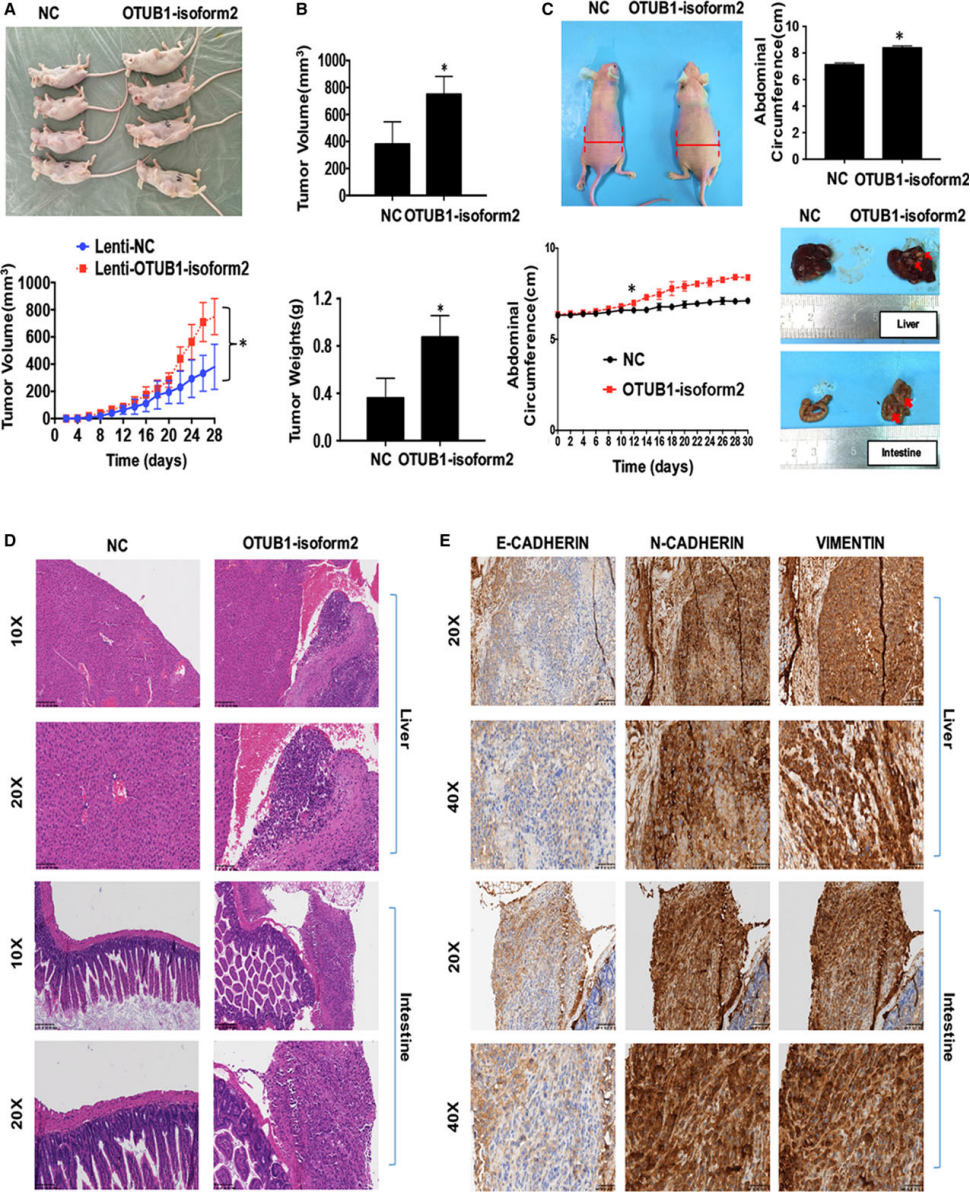
OTUB1‐isoform2 promotes ovarian tumour growth and metastasis in vivo. A, Tumour volumes of xenograft models were measured every 2 d. Photographs of nude mice with tumours are shown. *P <* *.01. B, The sizes and weights of the xenografts were compared between OTUB1‐isoform2 and NC groups. Analytical graphs were shown. *p <* *.01. C, Left: The representative images of nude mice. The abdominal circumferences were measured every 2 d and analysed. *from the 12th day on, *P *<* *.01. Right Top: The abdominal circumferences were compared between OTUB1‐isoform2 and NC groups. Analytical graphs were shown. *P <* *.01. Right Bottom: The representative images of intraperitoneal metastatic tumours in both NC and OTUB1‐isoform2‐overexpressing groups. The implanted lesions on the surface of livers and intestines were shown. Red arrows denoted the tiny implanted metastatic lesions. D, H&E staining was used to verify the formation of metastatic xenografts implanted on the surface of livers and intestines in the nude mice (top scale bars = 200 μm; bottom scale bars = 100 μm). E, Immunochemical staining was used to verify the expressions of E‐CADHERIN, N‐CADHERIN and VIMENTIN proteins in the peritoneal metastatic xenografts in the OTUB1‐isoform2‐overexpressing nude mice (top scale bars = 100 μm; bottom scale bars = 50 μm)

Finally, we used nude mouse xenograft and intraperitoneal metastasis models to investigate the biological functions of OTUB1‐isoform2 in vivo. Overexpression of OTUB1‐isoform2 accelerated the growth of subcutaneous xenografts when compared with the NC controls (*P *<* *.01, Figure 6A). The sizes and weights of xenografts in the OTUB1‐isoform2‐overexpressing group were significantly larger than those in the NC group (*P *<* *.01, Figure 6B). The intraperitoneal metastasis models showed that from the 12th day, the abdominal circumference was significantly, rapidly increased in the OTUB1‐isoform2‐overexpressing group compared with that in the NC group (*P *<* *.01, Figure 6C Left). Till the 30th day, the abdominal circumference in the OTUB1‐isoform2‐overexpressing group (8.39 ± 0.09 cm) was significantly larger than that (7.11 ± 0.08 cm) in the NC group (*P *<* *.01, Figure 6C Right Top). Tiny tumour nodules were formed and implanted on the surface of the liver, intestines and peritoneal membranes in the OTUB1‐isoform2‐overexpressing group but not in the NC group (Figure 6C Right Bottom and D). Moreover, immunochemistry experiments revealed that N‐CADHERIN and VIMENTIN were highly and strongly expressed in the lesions where E‐CADHERIN showed only weak and focal expression (Figure 6E). These data suggest that OTUB1‐isoform2 promotes ovarian tumour growth and metastasis in vivo.

## 4 DISCUSSION

In this study, we identified the expression of the ncRNA OTUB1‐isoform2 in the paired ovarian tumour and paratumorous tissues for the first time. The mRNA level of OTUB1‐isoform2 was increased in the tumour tissues compared with that in the paired paratumorous tissues. In the tumour tissues, high expression of OTUB1‐isoform2 was correlated with the FIGO stage and invasion‐related parameters such as vascular invasion, fallopian tubes involvement, extensive omentum involvement and lymph node metastasis. High expression of OTUB1‐isoform2 predicted poorer DFS and DSS rates. In vitro experiments also suggested that OTUB1‐isoform2 promoted ovarian tumour cell proliferation and invasion. Finally, the in vivo xenograft models supported the idea that OTUB1‐isoform2 accelerated tumour growth speeds, promoted greater tumour size and weight and facilitated intraperitoneal metastasis. These data suggested that OTUB1‐isoform2 predicted poor prognosis and promoted tumour progression in ovarian cancer.

The OTUB1 gene has two isoforms and the isoform 1 is the predominant transcript that encodes the deubiquitinase OTUB1. OTUB1 is abnormally up‐regulated in somatic malignancies and exerts oncogenic functions.^26^, ^32^, ^33^, ^34^ We have also previously demonstrated that OTUB1 prompted tumour growth and metastasis in ovarian cancer and gastric carcinoma.^25^, ^29^ In contrast, OTUB1‐isoform2 has been neglected for a long time because it has been regarded as an ncRNA in somatic organs except in the lymphatic tissues,^20^ and ncRNAs were once considered functionless.^7^, ^35^ Another reason for this negligence might be that the expression level of OTUB1‐isoform2 is several times lower than the predominant isoform OTUB1 in normal tissues.^20^, ^27^

However, with growing evidence revealing the essentiality of ncRNAs in biological functions and mechanisms, the importance of OTUB1‐isoform2 needs further evaluation. Our previous study^27^ and the present one found that despite its relatively low expression level in normal tissues, OTUB1‐isoform2 expression was significantly increased in tumour tissues and was correlated with advanced stage and invasion‐related indexes. Moreover, these clinical analyses were supported by both in vitro experimental data and in vivo models. These data suggested that OTUB1‐isoform2 was not a functionless isoform, but rather essential to tumorigenesis in gastric, ovarian and probably other somatic malignancies.

One concern is whether OTUB1‐isoform2 exerts these functions independently of its predominant isoform OTUB1. Previous studies have revealed that different isoforms of one gene could have distinct and exclusive functions similar to the transcriptional suppressor FOXM1a and its isoforms (b and c) which act as the transcriptional activators.^36^, ^37^, ^38^ To solve this problem, we performed the RNA gel electrophoresis and the subsequent sequencing of the PCR products of specific primers for these two isoforms. We also analysed the correlation between their expression and designed overexpression plasmids to exclude mutual interference. All these data suggested that the expression and functions of OTUB1‐isoform2 were independent of the predominant isoform OTUB1. This finding might be of vital importance, as OTUB1‐isoform2 might escape from the attack of molecular drugs that are designed only to target OTUB1, and then continue to be functional. Future pharmacological studies need to take both isoforms into account to ensure drug efficiency.

In 2004, Soares et al found that in lymphatic tissues, OTUB1‐isoform2 could encode the protein otubain 1 alternative reading frame 1 (ARF‐1), which has an opposing function to that of OTUB1 in controlling the stability of GRAIL expression to make T cells functionally anergic. In our studies, OTUB1‐isoform2 exerts oncogenic functions in gastric^27^ and ovarian cancers which is consistent with its isoform OTUB1.^25^, ^29^ The possible reasons for this are as follows: in the immune system, OTUB1‐isoform2 might function by encoding protein ARF‐1 to balance the functions of OTUB1 in normal T cells, thus regulating the CD4 T cell anergy. But in our studies, OTUB1‐isoform2 functions in the form of a ncRNA in the tumour tissues where the normal balancing mechanisms are dysregulated. Therefore, it is likely that these two isoforms function cooperatively in tumours. Future studies might focus on the potential interaction between these two isoforms on common target genes.

Finally, we found that OTUB1‐isoform2 is an independent predictor of poor prognosis in ovarian cancer. Since it functions in the form of a ncRNA, it would be convenient to detect the level of OTUB1‐isoform2 level in the serum to monitor the prognosis of patients. Future studies could use larger serum samples to detect the mRNA level of OTUB1‐isoform2 and evaluate its clinical application.

In summary, our study suggested that OTUB1‐isoform2 promoted tumour progression independent of its predominant isoform OTUB1 and predicted a poor prognosis in ovarian cancer. Our findings may be of some help to other researchers by providing some new insights into the molecular details of a potential pharmacologic target in ovarian cancer.

## CONFLICTS OF INTEREST

No potential conflicts of interest were disclosed.
